# Geographical gradients of dissolved Vitamin B_12_ in the Mediterranean Sea

**DOI:** 10.3389/fmicb.2013.00126

**Published:** 2013-06-10

**Authors:** S. Bonnet, A. Tovar-Sánchez, C. Panzeca, C. M. Duarte, E. Ortega-Retuerta, S. A. Sañudo-Wilhelmy

**Affiliations:** ^1^IRD, MIO, UM 110 - IRD Centre of Noumea, Aix-Marseille University, University of South Toulon Var, CNRS/INSUNoumea, New Caledonia, France; ^2^Department of Global Change Research, IMEDEA (CSIC-UIB), Instituto Mediterráneo de Estudios AvanzadosEsporles Balearic Islands, Spain; ^3^Department of Biological Sciences, University of Southern CaliforniaLos Angeles, CA, USA; ^4^The UWA Oceans Institute, The University of Western AustraliaCrawley, WA, Australia; ^5^Departament Biologia Marina i Oceanografia, Institut de Ciències del Mar, CSICBarcelona, Spain

**Keywords:** vitamin B_12_, Mediterranean Sea, growth factor, phytoplankton, bacteria

## Abstract

Most eukaryotic phytoplankton require vitamin B_12_ to grow. However, the cycling of this organic growth factor has received substantially less attention than other bioactive substances such as trace metals in the marine environment. This is especially true in the Mediterranean Sea, where direct measurements of dissolved vitamins have never been reported. We report here the first direct measurements of dissolved vitamin B_12_ across longitudinal gradients in Mediterranean waters. The range of vitamin B_12_ concentrations measured over the whole transect was 0.5–6.2 pM, which is slightly higher than the range (undetectable—4 pM) of ambient concentrations measured in other open ocean basins in the Pacific and Atlantic oceans. The concentrations measured in the western basin were significantly higher (*p* < 0.05) than those of the eastern basin. They were positively correlated with chlorophyll concentrations in the most western part of the basin, and did not show any significant correlation with any other biological variables in other regions of the sampling transect.

## Introduction

The Mediterranean Sea is an oligotrophic ecosystem (Mc Gill, 1965; Krom et al., 1991), with a west to east gradient of increasing oligotrophy. It is characterized by a strong thermal stratification with a sharp thermocline (10–20 m deep) during late spring to fall, and a mixing period in winter, leading to a phytoplankton bloom in the early Spring (e.g., Marty et al., 2002; Moutin et al., 2012).

Surface macro-nutrient concentrations also depend on the exchanges with the Atlantic Ocean (through the Strait of Gibraltar), the Black Sea (through the Bosphorus Strait and Marmara Sea), and depend on river discharge. In addition, the Mediterranean Sea receives the highest rate of aeolian dust deposition of the world's oceans (Guerzoni et al., 1999) as well as anthropogenic aerosols from industrial and domestic activities from the highly populated areas around the basin (Chester et al., 1996; Guieu et al., 1997). Consequently, Mediterranean surface waters exhibit relatively high dissolved iron (Fe) concentrations that are linked to the dynamics of atmospheric deposition and water column stratification (Bonnet and Guieu, 2006). Therefore, Fe availability rarely limits primary and prokaryotic heterotrophic production in those waters (Bonnet et al., 2005; Pulido-Villena et al., 2008). However, the elemental stoichiometry measured in different pools (i.e., particulate and dissolved, inorganic and organic) reveals a deficiency of phosphorus (P) relative to nitrogen (N) (Bethoux et al., 2002), and phosphorus (P) availability has been seen to limit primary production, prokaryotic heterotrophic production and N_2_ fixation along the Mediterranean basin (e.g., Thingstad and Rassoulzadegan, 1995; Lasternas et al., 2010; Ridame et al., 2011).

Besides inorganic bioactive elements such as Fe and P, most eukaryotic phytoplankton require organic nutrients such as vitamin B_12_ (Provasoli and Carlucci, 1974; Droop, 2007). Vitamin B_12_ is a cobalt-containing organometallic compound involved in several vital enzymes in the central metabolism of algae (Raux et al., 2000; Martens et al., 2002). Many eukaryotic phytoplankton lack the biosynthetic pathway for vitamin B_12_. Croft et al. (2005, 2006) recently showed that over 50% of the 326 algal species tested in culture collections were unable to grow without any vitamin B_12_ additions, showing that they have an absolute requirement for this co-factor and thus depend on an exogenous pool. In the open and coastal ocean, the ambient pool may be insufficient to support maximum primary productivity as vitamin B_12_ amendments have been shown to stimulate phytoplankton growth in the Atlantic, Pacific and Southern Ocean (Panzeca et al., 2006; Sañudo-Wilhelmy et al., 2006; Bertrand et al., 2007; Gobler et al., 2007; Koch et al., 2011). The ambient pool of vitamin B_12_ depends upon prokaryots (Archaea, Bacteria, Guillard, 1968) as they possess the biosynthetic pathway to produce this vitamin, and eukaryotic algae would acquire vitamin B_12_ either from symbiotic bacteria or directly from the dissolved pool (Provasoli, 1963; Croft et al., 2005; Droop, 2007). Furthermore, vitamin concentrations have been linked to shifts in plankton community composition (Koch et al., 2011). Recent laboratory studies also showed that ubiquitous picocyanobacteria, such as *Synechococcus* and unicellular diazotrophic cyanobacteria such as *Crocosphaera*, were also able to produce and release vitamin B_12_, with higher production rates compared to heterotrophic bacteria (Bonnet et al., 2010).

Despite the recognized biogeochemical importance of vitamin B_12_ in the Ocean, the cycling of this organic growth factor has received substantially less attention than other bioactive substances such as trace metals in the marine environment. This is especially true in the Mediterranean Sea, where direct measurements of dissolved vitamins have never been reported. Measuring vitamin B_12_ concentrations in seawater represents a technical challenge, as this cofactor is found at picomolar (10^−12^ M) levels in open ocean waters (e.g., Panzeca et al., 2008, 2009). The development of direct and efficient methods, in contrast to the indirect microbiological assays, to measure low levels of dissolved vitamin B_12_ (Okbamichael and Sañudo-Wilhelmy, 2004) now allows us to expand our knowledge on the biogeochemical cycling of this growth factor in the ocean (e.g., Panzeca et al., 2006, 2008, 2009; Sañudo-Wilhelmy et al., 2006, 2012; Suárez-Suárez et al., 2011).

**Figure 1:**
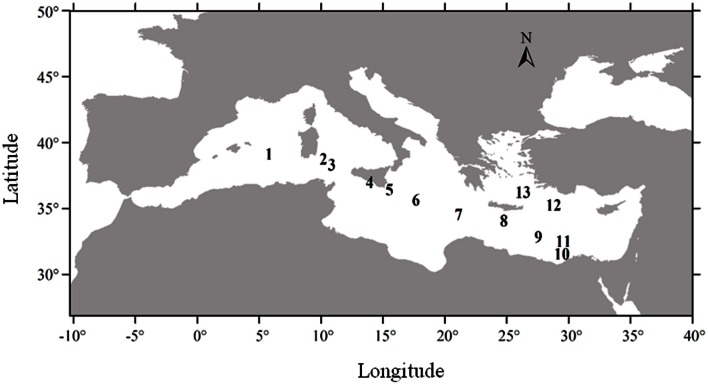
**Location of the thirteen stations of the cruise**.

In late spring 2007, we measured dissolved vitamin B_12_ concentrations along a 3000 km west-east transect in the Mediterranean Sea (Figure 1), that exhibited nutrient and chlorophyll gradients, to characterize the vertical distribution of vitamin B_12_ concentrations along with other relevant hydrological and biogeochemical factors.

## Materials and methods

Dissolved water samples for vitamin B_12_ and other biological and chemical parameters were collected during May 2007 along a longitudinal transect in the Mediterranean Sea onboard the Spanish ship R/V Garcia del Cid. A total of 13 stations were sampled across a west-to-east transect (Figure 1), starting southwest of Sardinia and finishing in the Aegean Sea (Station 13). The cruise track also covered the eastern Mediterranean basin, near the Egyptian coast (Station 10).

### Hydrological and biogeochemical measurements

Vertical profiles of temperature and salinity were obtained using a Seabird 911 plus CTD. Seawater samples were collected at 6 depths between the surface and 200 m depth using 12 l Niskin bottles mounted on a rosette sampler.

Chlorophyll *a* (Chl *a*) concentrations were determined fluorometrically according to the method of Parsons et al. (1984). At each depth, 50 ml of seawater were filtered through 25 mm glass fiber filters (Whatman GF/F), extracted into 10 ml of 90% acetone for 24 h in the dark at 4°C. The fluorescence of the extracts was then measured on a calibrated Turner Designs fluorometer (Parsons et al., 1984).

For inorganic nutrients (nitrate, phosphate, silicate), samples were collected in acid-washed 20 ml plastic flasks. Concentrations were determined using standard colorimetric techniques on a Bran Luebbe autoanalyser AA3. Detection limits for the procedures were 0.05 μM, 0.01 μM, and 0.1 μM for NO^−^_2_+NO^−^_3_, PO^3−^_4_ and Si(OH)_4_, respectively.

### Vitamin B_12_ concentration measurements

At each depth, 2 l of seawater were sampled for dissolved vitamin B_12_ concentration determination. Briefly, immediately after collection, samples were acidified to pH 6 using 12 N trace metal grade HCl, transferred to sterile 2 l intravenous (IV) bags, and pumped through 5 g of Bondesil C18 resin (pre-conditioned with methanol) at a flow rate controlled at 1 ml min^−1^. Columns were then rinsed with 20 ml of MilliQ water and eluted with 5 ml HPLC grade methanol. The eluent was then evaporated under vacuum (Labconco Rapid-Vac), redissolved in 200 μ l of MilliQ water, and analyzed by High Performance Reverse Phase Liquid Chromatography (Shimadzu 10AD-vp) according to Okbamichael and Sañudo-Wilhelmy (2004). Samples were filtered again through 0.45 μm small-volume syringe filters (Millex®- FH) to further purify the sample before injection into the HPLC The method and filter type was tested using B_12_ standard recovery (Panzeca, 2007).

### Prokaryotic abundances (PA) and prokaryotic heterotrophic production (PHP) measurements

PA was determined by flow cytometry according to Trousselier et al. (1995) after fixation of samples (4 ml) using a mixture of paraformaldehyde and glutaraldehyde (1%) and freezing in liquid nitrogen. Briefly, 200 μl of each sample was stained with 4 μl of 5 μmol l^−1^ SYBR Green (Molecular Probes) for 10 minutes in the dark, and run through a FACS calibur™ flow cytometer (BD Biosciences) fitted with a laser emitting at 488 nm. Samples were run at a low flow rate and data were acquired in log mode until around 10,000 events were acquired. A stock solution (5 μl) of yellow–green 0.92 μm Polysciences latex beads was added as an internal standard per 200 μl of sample. The concentration of the fluorescent beads was calibrated every 2 days by direct microscope enumeration. Prokaryotic cells were detected by their signature in bivariate plots of side scatter (SSC) vs. green fluorescence (FL1). Data were gated and counted in the SSC vs. FL1 plot using the Paint-a-Gate software (del Giorgio et al., 1996; Gasol and Del Giorgio, 2000). PA was expressed in cells per liter.

PHP was estimated from 3H-leucine–protein synthesis following the microcentrifugation technique described in Smith and Azam (1992). Briefly, 5 μl of L-[4,5-3H] leucine were added to 1.5 ml samples, yielding a final concentration of 28.8 nM, and were incubated for 3 h. Incubations were stopped by addition of trichloroacetic acid (5% final concentration) and samples were stored at −20°C until processing at the home laboratory. We used a conversion factor from leucine to carbon incorporation of 1.5 kg C mol leu^−1^ (Simon and Azam, 1989).

Ocean Data View software was used for graphical presentation of all the data mentioned above. For constructing of the charts, one of the ODV interpolation methods—VG Gridding (X and Y scale length: 150) was used.

### Statistics

Vitamin B_12_ concentrations in the eastern and western basins were compared using a 2-tailed non parametric mean comparison test (*n* = 3, α = 0.05, unpaired samples). Surface (0–100 m) and deep (100–200 m) vitamin B_12_ concentrations were compared using a 2-tailed non parametric mean comparison test (*n* = 3, α = 0.05, paired samples). To examine the relationship between vitamin B_12_ concentrations, PA, PHP, and Chl *a* concentrations, Pearson's correlation coefficients were calculated and tested between each variable of interest (degree of freedom = *n* − 2, α = 0.05).

## Results

### Hydrological and biogeochemical background conditions

**Figure 2:**
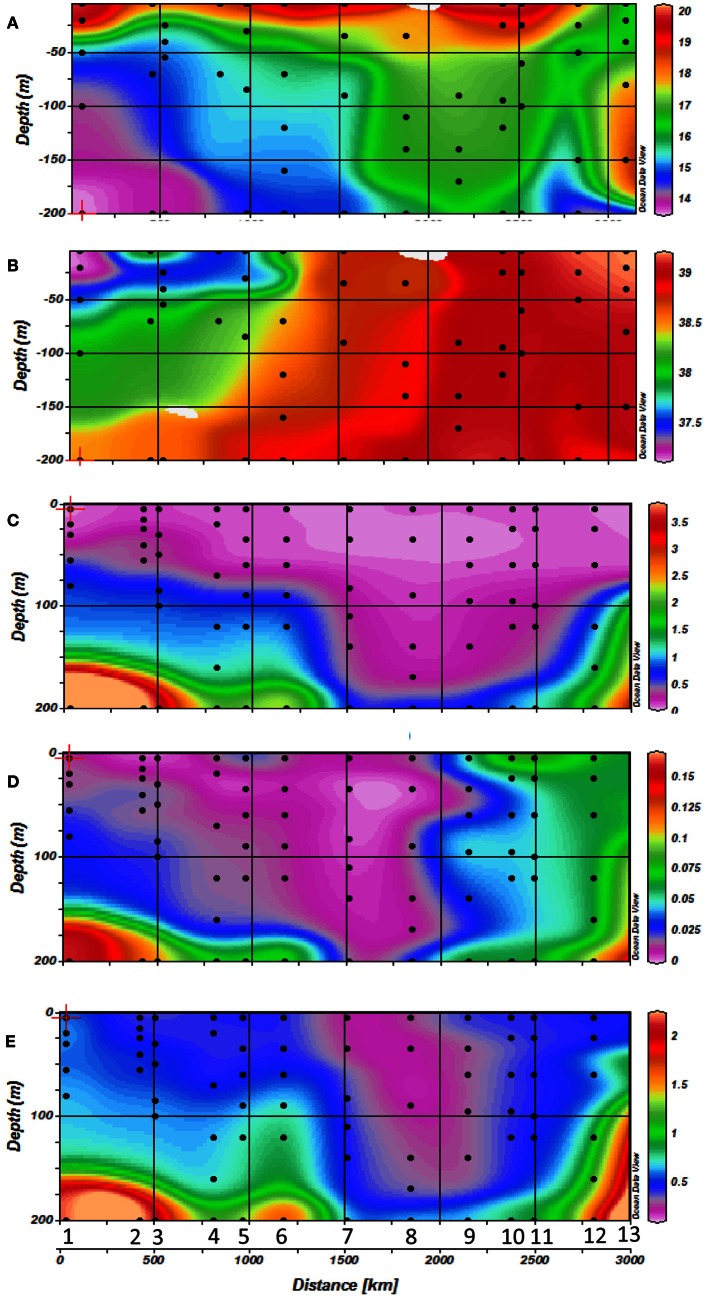
**Horizontal and vertical distribution of **(A)** temperature, **(B)** salinity, **(C)** NOx concentrations, **(D)** PO_4_ concentrations and **(E)** Silicate concentrations along the transect with station numbers (1–13) and sampling depths indicated by dots during cruise**.

Temperature ranged from 15.95–20.24°C across the Mediterranean basin over the first 200 m (Figure 2A). These figures show the occurrence of thermal stratification along the whole transect, but with a shallower thermocline depth in the western basin (around 40 m depth) compared to the eastern basin (50–200 m depth). There was also a strong horizontal salinity gradient from west to east with a marked halocline in the Ionian Sea (stations 5 and 6) (Figures 1, 2B). This gradient separates western Atlantic waters entering the Mediterranean Sea through the Strait of Gibraltar, from the high salinity waters of the eastern Mediterranean Sea. Salinity was lower on the western side of the transect (down to 37 in surface waters), and gradually increased eastwards to reach up to 39 in the top 200 meters.

Surface waters were depleted in nutrients (Figures 2C–E). NO^−^_2_ + NO^−^_3_ (hereafter NO_x_) concentrations were low (around 0.1 μM) along the whole transect. The thickness of this depleted layer increased towards the east from about 50–80 m in the western part of the transect to more than 180 m in the eastern basin. Phosphate concentrations followed the same geographical trend as NO_x_, with surface concentrations close to the detection limit of conventional micromolar methods (0.01 μM), and a progressive deepening of the phosphacline going eastward. Surface phosphate concentrations increased in the Aegean Sea (stations 12 and 13) to reach around 0.06–0.07 μM. The NO_x_:PO_4_ molar ratio was 21 over the whole cruise suggesting that the Mediterranean was potentially phosphate limited with respect to nitrate during our sampling campaign.

Silicate concentrations decreased from west to east to reach up to 0.18 μM in the eastern basin (Figure 2E), and increased again to 0.5 μM in surface waters of the Aegean Sea (stations 12 and 13). As seen with NO_x_ and PO_4_, the depth of the silicacline deepened in the east (Figures 2C–E). The average stoechiometric Si:NO_x_ molar ratio was 2.5 over the whole transect, which suggested that silicate was in sufficient supply relative to nitrate.

**Figure 3:**
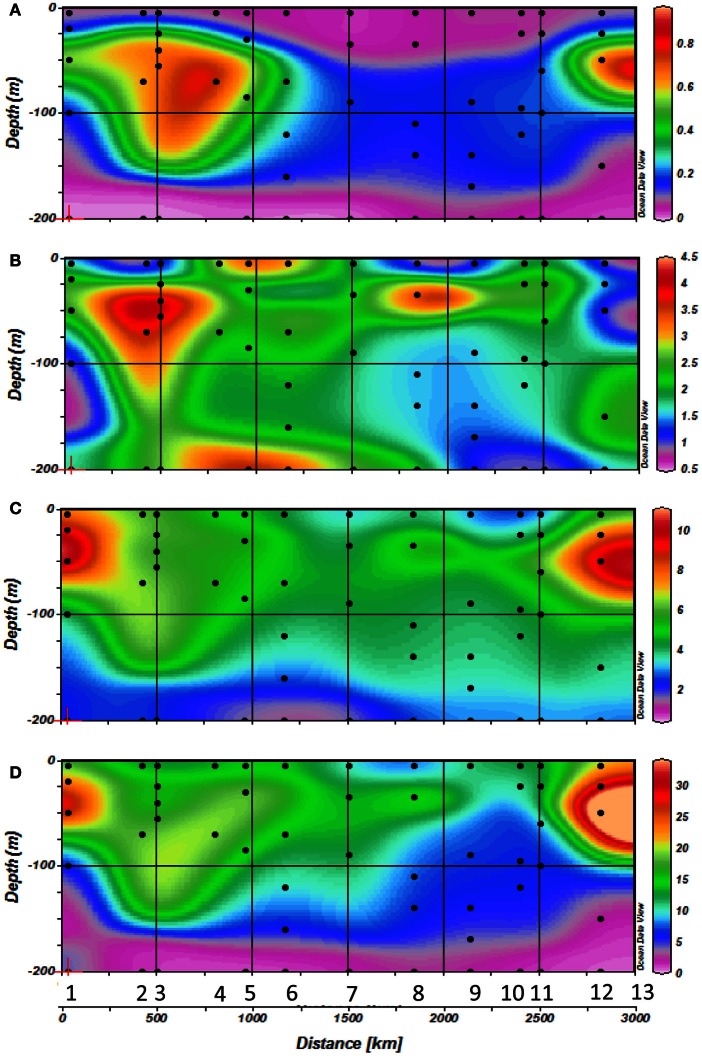
**Horizontal and vertical distribution of **(A)** Vitamin B_12_ concentrations, **(B)** Chlorophyll *a* concentrations, **(C)** Prokaryotic abundances (PA) and **(D)** Prokaryotic heterotrophic production (<php) rates along the transect with station numbers (1–13) and sampling depths indicated by dots during cruise**.

Chl *a* concentrations (Figure 3A) ranged from 0.02 to 1.6 μg l^−1^ over the 0–200 m layer of the studied transect. The whole section was characterized by a deepening of the Deep Chlorophyll Maximum (DCM) from west (40–80 m) to east (80–125 m), associated with the increasing oligotrophy. Chl *a* concentrations in the DCM also decreased eastward. In the last 2 stations of the transect located in the Aegean Sea (stations 12 and 13), Chl *a* concentrations increased to 0.97 μg Chl *a* l^−1^ in the DCM, and the depth of the DCM was shallower (around 60 m) as in the western side of the Mediterranean.

### Vitamin B_12_ concentrations

**Figure 4:**
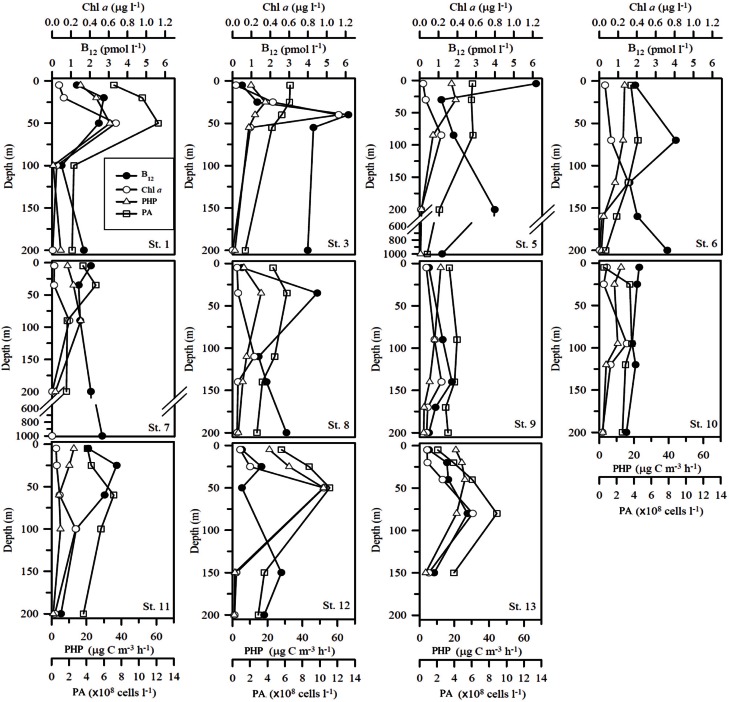
**Vertical profiles of vitamin B_12_ (pM), Chl *a*, Prokaryotic abundances (PA) and Prokaryotic heterotrophic production (PHP)**. Units for B_12_ and Chl *a* concentrations, PA and PHP are pmol l^−1^, μg l^−1^, ×10^8^ cells l^−1^ and μg C m^−3^ h^−1^, respectively. Note that profiles for stations where only two data points were available are not plotted as vertical profiles.

The range of vitamin B_12_ concentrations (Figures 3B, 4) measured over the Mediterranean transect was 0.5–6.2 pM. A slight longitudinal gradient was present; the concentrations measured in the western basin (stations 1–6: 2.44 ± 1.64 pM; mean of all profiles from the western basin ± standard deviation) were significantly higher (*p* < 0.05) than those of the eastern basin (stations 7–13: 1.67 ± 0.92 pM) (2-tailed non parametric mean comparison test). Maximum values were reached at 40 m depth at station 3 (6.15 pM) and in surface at station 5 (6.20 pM). Most vertical profiles exhibited maximum vitamin B_12_ concentrations close to the DCM (Figure 4), except at station 5. Other B_12_ depth profiles were more homogeneous vertically (Stations 7 and 10). The two stations where samples were collected at 1000 m (i.e., stations 5 and 7) exhibited concentrations of 1.19 and 2.7 pM at this depth, respectively.

### Prokaryotic abundances and production

Prokaryotic abundance (Figure 3C) declined markedly from west to east. Maximum abundances were measured at station 1 at 20-50 m depth with 12 × 10^8^ cells l^−1^. The abundance maxima then decreased towards the east and was located deeper within the water column (around 75 m depth). Abundances increased again at the Aegean Sea at the end of the transect to reach concentrations around 11 × 10^8^ cells l^−1^ at station 12 at 50 m depth.

Prokaryotic heterotrophic production (Figure 3D) followed approximately the same trend observed for prokaryotic abundance with maximum rates measured at station 1 at 20–50 m depth (25–33 μg C m^−3^ h^−1^); it then decreased toward the eastern basin to reach minimum rates at stations 10 and 11 (10–12 μg C m^−3^ h^−1^), increasing again in the Aegean Sea, reaching up to 52 μg C m^−3^ h^−1^.

## Discussion

The Mediterranean Sea displayed a large variety of hydrological conditions during the stratification period, covering a large range of trophic conditions from the oligotrophic western basin to the ultra-oligotrophic eastern basin (Figures 2, 3). The west-to-east gradient in hydrological and biogeochemical conditions encountered during our cruise (thermal stratification, nutrient depletion in surface waters and the deepening of the nutriclines and the DCM) are typical conditions for the Mediterranean Sea during the so-called stratification period (e.g., Moutin et al., 2012).

Vitamin B_12_ is known to be a labile organic molecule with a short half-life (days) in seawater (Carlucci et al., 1969). Thus, elevated concentrations are generally associated with local production. Bacteria are the primary producers of this organic growth factor (Raux et al., 2000; Martens et al., 2002), but as soon as it is produced, it is consumed by eukaryotic phytoplankton and some ubiquitous heterotrophic bacteria lacking biosynthetic pathways for vitamin B_12_ (Giovannoni et al., 2005; Bertrand et al., 2007). The relation between production and stocks is thus sometimes more complex. In this study, vitamin B_12_ distributions do not show any significant correlation with spatial patterns observed for prokaryotic heterotrophic production and abundance (Figure 3, *r* = 0.04 and −0.04 respectively, *p* > 0.05), probably because vitamin B_12_ stocks are the net result of vitamin production and consumption by micro-organisms.

The depth distributions of vitamin B_12_ were correlated to the chlorophyll maximum in the western part of the transect (stations 1–3, *r* = 0.66, *p* < 0.05). This trend is contrary to what has been observed in some eutrophic coastal systems (Sañudo-Wilhelmy et al., 2006) where chlorophyll stocks in the fraction >5 μm were inversely correlated with vitamin B_12_ concentrations; these inverse distributions were interpreted as vitamin consumption by large size phytoplankton species in these coastal areas, as most large phytoplanktonic species are auxotrophic for vitamin B_12_ (Croft et al., 2005, 2006). In the Mediterranean Sea, during the stratification period, large-size phytoplankton are scarce and the system is dominated by prokaryotic phytoplankton which are pico-cyanobacteria *Synechococcus* and *Prochlorococcus* (Marty et al., 2002; Lasternas et al., 2010). Contrary to eukarytotic phytoplankton who are B_12_ consumers, recent studies indicate that *Synechococcus* is able to produce and excrete large amounts of vitamin B_12_ (Bonnet et al., 2010), and *Prochlorococcus* also possess the biosynthetic pathway for vitamin B_12_ production. Cyanobacterial counts are not available for this study but recent studies indicate that they are maximum at the DCM in Mediterranean waters during the season studied (Mella-Flores et al., 2011); vitamin B_12_ production by these cyanobacteria may explain the large vitamin B_12_ stocks measured at stations 2 and 3 in the DCM. It has to be noted that relatively high vitamin B_12_ concentrations were also measured in surface waters above the DCM at stations 4 and 5. These concentrations may be attributed either to prokaryotic heterotrophic production, or to synthesis by small cyanobacteria located in surface waters. Recent studies have reported an unusual near surface *Synechococcus* abundance maximum close to the Sicily Strait (Mella-Flores et al., 2011) during the stratification period in the Mediterranean Sea, which might explain high production rates of vitamin B_12_. This cyanobacterial maximum is unusual compared to other oligotrophic areas (Olson et al., 1988; Li et al., 1992) and has been attributed to surface nutrient enrichment by atmospheric dust deposition (Mella-Flores et al., 2011). In fact, it has been shown that dust inputs cause an increase predominantly in the cyanobacteria in Mediterranean waters (Bonnet et al., 2005). At stations 4 and 5, nutrient inputs could also be from land sources as those stations are located near the Sicilian coast and usually that region is less oligotrophic than the rest of the Mediterranean Sea (e.g., Moutin et al., 2012). This hypothesis is consistent with the slight increase in phosphate concentrations observed in the present study (Figure 2).

Vitamin B_12_ concentrations were lower in deeper waters (100–200 m) compared to surface and subsurface (0–100 m) waters (*p* < 0.05), except at stations 3, 5, and 6 where they reached 3.99, 3.99, and 3.51 pM, respectively at 200 m depth. Stations 5 and 6 are located close to the Sicily strait, which separates the Mediterranean eastern and western basins, leading to active and complex hydrodynamic features (Pinardi and Masetti, 2000); we could hypothesize that vitamin B_12_ has been upwelled from deeper waters, potentially explaining the maxima seen at those stations. The two stations for which data are available below 200 m (stations 5 and 7) show concentrations of 1.19 and 2.67 pM at 1000 m, which are not very high but the lack of data between 200 and 1000 m makes it difficult to interpret. The only report of deep vitamin B_12_ concentrations (surface to 800 m depth) available (Sañudo-Wilhelmy et al., 2012) indicate that maximal concentrations were found in the upper mesopelagic zone, around 300 m and surface maxima can be the result of vitamin transport by different water masses. In the Mediterranean Sea, future studies including a deeper vertical resolution are needed to better understand B_12_ distributions.

Depth-averaged vitamin concentrations were significantly (*p* < 0.05) higher in the western basin (2.44 ± 1.64 pM) and decreased slightly going eastward towards more oligotrophic waters (1.67 ± 0.92 pM). This trend is consistent with the survey performed by Moschopoulou and Ignatiades (1993), who measured the seasonal and spatial distribution of vitamin B_12_ in the Saronicos Gulf (Aegean Sea). They reported concentrations ranging between 0.8 and 5.8 pM, which are higher than those measured in the present study within the same area (1.39 ± 1.79 pM). However, those results were obtained indirectly using the microbiological assay method that may not accurately reflect ambient conditions, as they were also conducted in 0.45–0.8 μm filtered seawater that may have included B-vitamin–producing bacteria in the filtrate (Menzel and Spaeth, 1962; Carlucci and Bowes, 1970). Despite the few vitamin B_12_ data available in Mediterranean waters, the west-to-east gradient of B_12_ observed in our study is consistent with previous studies. For example, Martin and Vacelet (1975) and Fiala (1982) reported higher values in the western basin (French coast) compared to those reported for the eastern basin by Moschopoulou and Ignatiades (1993).

**Figure 5:**
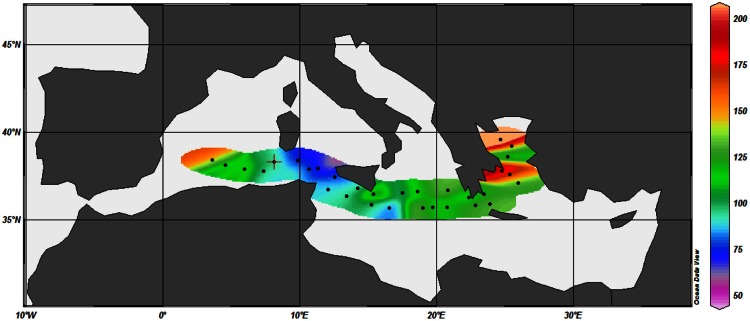
**Surface dissolved cobalt concentrations (pM) along the Mediterranean transect**. Surface seawater (<0.22 μm) was collected during the cruise THRESHOLDS I (from June 5 to June 30, 2006; 35.6–41.9°N, 3.6–30.1°E) according to (Tovar-Sánchez, 2012). Cobalt was pre-concentrated by the APDC/DDDC organic extraction method and analyzed by ICP-MS according to protocols described in (Tovar-Sánchez, 2012).

The overall range of vitamin B_12_ concentrations measured in the Mediterranean Sea in the present study (0.5–6.2 pM) are slightly higher than the range of ambient concentrations (undetectable—4 pM) measured in other open ocean locations in the world ocean (Table 1). For example, vitamin B_12_ concentrations at stations located off the coast of Baja California were undetectable for 100 s of kilometers (Sañudo-Wilhelmy et al., 2012). In the Southern Ocean, the North Atlantic and the South Indian Ocean, vitamin B_12_ concentrations ranged from 0.4 to 4 pM (Panzeca et al., 2009), 0.1–2.5 pM (Panzeca et al., 2008) and 0.1–3 pM (Fiala and Oriol, 1984) respectively (Table 1). The reasons for such relatively high B_12_ concentrations in Mediterranean waters despite oligo- to ultra-oligotrophic conditions are unclear. We can hypothesize that vitamin B_12_ accumulates in surface waters because the growth of the main consumers (eukaryotic phytoplankton) is limited by macronutrients (nitrate and phosphate, e.g., Lasternas et al., 2010; Ridame et al., 2011; Tanaka et al., 2011) in this area. Another hypothesis is that the high cobalt concentrations in Mediterranean waters stimulate de novo synthesis of vitamin B_12_ as cobalt is the central metal ion in the B_12_ molecule (Raux et al., 2000; Martens et al., 2002). Panzeca et al. (2008, 2009) have shown that the spatial distribution of vitamin B_12_ in various coastal and open ocean waters followed the abundance of total dissolved cobalt. Surface dissolved cobalt concentrations measured in surface Mediterranean waters are high (45–291 pM, Figure 5 and Heimbürger et al., 2009) compared to those of the Atlantic (5–87 pM, Saito and Moffett, 2002; Panzeca et al., 2008) and the Pacific Ocean (30–105 pM, Knauer et al., 1982). They have been attributed to the high atmospheric dust inputs (Heimbürger et al., 2009) as cobalt is a crustal constituent of dust (1.8%) and is soluble in seawater (Thuróczy et al., 2010). Future studies combining simultaneous analysis of dissolved cobalt and vitamin B_12_ will be necessary to substantiate their possible relationship in Mediterranean waters.

**Table 1:** **Ranges of vitamin B_12_ concentrations reported in different marine areas of the world**.

Studied area^*^	**Range of vitamin B_12_ concentrations (pmol l^−1^)**	**References**
Sargasso Sea	0–0.3	Menzel and Spaeth (1962)
San Pedro Basin, California, USA	0.2–1.8	Panzeca et al. (2009)
North Atlantic surface waters	0.1–2.5	Panzeca et al. (2008)
Northeast Pacific Ocean	0–2.7	Carlucci and Silbemagel (1966)
Southern part of the Indian Ocean	0.1–3.0	Fiala and Oriol (1984)
Bay of Biscay	0.1–3.7	Daisley and Fisher (1958)
Gerlache Strait, Southern Ocean	0.4–4	Panzeca et al. (2009)
Mediterranean Sea	0.5–6.2	This study

* *The studied areas are ranked by the high limit of reported vitamin B_12_ concentration range*.

This study reports the first direct measurements of dissolved vitamin B_12_ concentrations across longitudinal and trophic gradients in Mediterranean waters. Because vitamin B_12_ is an essential nutrient for most algal species (Croft et al., 2005), the availability of this growth factor could play a significant role on phytoplankton successions and carbon export in Mediterranean waters. Whereas our study could not resolve the possibility of B_12_ limitation in the Mediterranean, it did provide insights onto the possible mechanisms influencing the geographical distribution of vitamin B_12_. Future studies will need to address the factors, such as photochemical degradation, production, excretion, and uptake rates by microorganisms, responsible for the cycling of vitamin B_12_ in Mediterranean waters.

### Conflict of interest statement

The authors declare that the research was conducted in the absence of any commercial or financial relationships that could be construed as a potential conflict of interest.
